# Prevalence of cesarean scar disorder in patients 3 years after a first cesarean section

**DOI:** 10.1111/aogs.70005

**Published:** 2025-08-21

**Authors:** Saskia J. M. Klein Meuleman, Carry Verberkt, Pere N. Barri, Ally Murji, Oliver Donnez, Grigoris Grimbizis, Ertan Saridogan, Tom Bourne, Jian Zhang, Michal Pomorski, Shunichiro Tsuji, Thierry van den Bosch, Sanne I. Stegwee, Judith A. F. Huirne, Robert A. de Leeuw, 2Close study group, CSDi study group

**Affiliations:** ^1^ Department of Obstetrics & Gynecology Amsterdam University Medical Center, Location Vrije Universiteit Amsterdam Amsterdam the Netherlands; ^2^ Amsterdam Reproduction and Development Amsterdam the Netherlands; ^3^ Department of Obstetrics, Gynecology and Reproductive Medicine Hospital Universitario Dexeus Barcelona Spain; ^4^ Department of Obstetrics and Gynaecology University of Toronto, Mount Sinai Hospital Toronto Ontario Canada; ^5^ Complex Endometriosis Center, Polyclinique Urbain V, (Elsan Group) Avignon France; ^6^ 1st Department of Obstetrics and Gynaecology Medical School, Aristotle University of Thessaloniki Thessaloniki Greece; ^7^ Reproductive Medicine Unit Elizabeth Garrett Anderson Wing Institute for Women's Health, University College Hospital London UK; ^8^ Institute for Reproductive and Developmental Biology, Imperial College London London UK; ^9^ Department of Development and Regeneration Katholieke Universiteit Leuven Leuven Belgium; ^10^ Department of Obstetrics and Gynecology International Peace Maternity and Child Health Hospital, School of Medicine, Shanghai Jiao Tong University Shanghai China; ^11^ 2nd Department of Gynaecology and Obstetrics Wroclaw Medical University Wroclaw Poland; ^12^ Department of Obstetrics and Gynecology Shiga University of Medical Science Otsu Shiga Japan; ^13^ Department of Obstetrics and Gynecology University Hospital KU Leuven Leuven Belgium; ^14^ Department of Obstetrics & Gynecology Erasmus Medical Center Rotterdam the Netherlands

**Keywords:** abnormal uterine bleeding, cesarean scar defect, cesarean section, chronic pelvic pain, infertility, postmenstrual spotting, uterine niche

## Abstract

**Introduction:**

A symptomatic uterine niche is a long‐term complication after a cesarean section (CS). A group of international niche experts reached consensus on a standardized definition of a disorder caused by a symptomatic niche, named cesarean scar disorder (CSDi). However, the prevalence of this disorder is unclear. The aim of this study was to assess the prevalence of CSDi in patients 3 years after a first CS.

**Material and Methods:**

A secondary analysis was performed on the 3‐year follow‐up results of the 2Close study. The 2Close study was a multicenter randomized controlled trial that evaluated single‐ versus double‐layer uterine closure at CS in 32 hospitals in the Netherlands and included 2292 patients (registered in Dutch trial register: [NTR5480]). Patients, aged ≥18 years, undergoing a first CS were included. Three months after their CS, transvaginal ultrasonography was performed to evaluate the uterine scar for the presence of a niche. Three years after their CS, a digital questionnaire was sent to evaluate the primary and secondary symptoms of CSDi. For this secondary analysis, patients were excluded if they were pregnant, breastfeeding, or using hormonal contraception. The primary outcome of the study was the prevalence of CSDi.

**Results:**

Of the 1648 participants who completed the 3‐year questionnaire, patients were excluded due to pregnancy or breastfeeding (*n* = 305), use of hormonal contraception (*n* = 509), missing ultrasound evaluations (*n* = 76), and incomplete responses (*n* = 88). Of the 670 patients included in this analysis, 543 (81.0%) had a uterine niche visible on ultrasound and 127 (19.0%) were without a niche. The prevalence of CSDi at 3 years following a first CS was 42.5% (285/670). Most reported symptoms were chronic pelvic pain (35.0%), postmenstrual spotting (32.8%), and abnormal vaginal discharge (23.2%).

**Conclusions:**

Our study found a high prevalence of CSDi 3 years following their first CS. Symptoms were self‐reported and the exclusion criteria of pregnancy, breastfeeding, or hormonal contraception use could have introduced selection bias. Therefore, this percentage could be an overestimation of the actual prevalence. However, this high prevalence should be included in counseling patients with a scheduled CS.

## Graphical abstract

Three years following a first caesarean section 42.5% of all patients develop Caesarean Scar Disorder. Most reported symptoms within the Caesarean Scar Disorder are chronic pelvic pain, postmenstrual spotting and abnormal vaginal discharge. The long‐term consequences of a Caesarean Section on patients’ health should be structurally investigated.

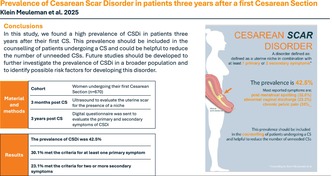

**Abbreviations**
CS: cesarean section
CSDi: cesarean scar disorder
TVUS: transvaginal ultrasonography

**Key message**

Three years following a first cesarean section, a high percentage of patients met the standardized definition for cesarean scar disorder, as a result of a symptomatic uterine niche. The most frequently reported symptoms were chronic pelvic pain (35.0%), postmenstrual spotting (32.8%), and abnormal vaginal discharge (23.2%).

## 1 INTRODUCTION

Recent studies by the World Health Organization indicated that by 2030, approximately a third (29%) of all births worldwide will take place by cesarean section (CS).^1^ Consequently, a corresponding increase in complications associated with CS can be expected. Well‐known complications after a CS are placenta accreta spectrum disorder, cesarean scar pregnancies, and the risk of a uterine rupture in a subsequent pregnancy.^2^, ^3^, ^4^ Furthermore, a CS is associated with reduced fertility and gynecological complaints such as spotting, dysmenorrhea, or chronic abdominal pain.^5^, ^6^

These complications are often attributed to inadequately healed myometrium at the site of the CS incision, also called a uterine niche.^7^, ^8^ In approximately 60% of individuals after a CS, a uterine niche is visible on transvaginal ultrasonography (TVUS) and almost 30% of the women with a niche experience symptoms.^9^, ^10^, ^11^

Recently, a group of international niche experts reached consensus on a disorder caused by a symptomatic uterine niche, named cesarean scar disorder (CSDi).^7^ This disorder is defined as a condition with several gynecological, reproductive, or psychosocial symptoms in the presence of a uterine niche confirmed by ultrasound.^8^

Due to the novelty of this disorder, the proportion of patients who develop CSDi after a CS still needs to be determined. Understanding this prevalence would be valuable for counseling patients before a CS and assisting them in making informed decisions. Therefore, this study aims to assess the prevalence of CSDi in patients 3 years after a primary CS.

## 2 MATERIAL AND METHODS

### 2.1 Study design

This study is a secondary analysis of a multicenter randomized controlled trial (2Close study) comparing single‐ with double‐layer uterine closure after a first CS. This study protocol and primary results have been published before.^5^, ^12^ Recently, 3‐year follow‐up results have also been published.^13^ The study was approved by the Institutional Review Board of AmsterdamUMC, location VUmc, and the boards of all participating hospitals before enrolment started. The study was registered in the Dutch trial register (NTR5480). This article adheres to the CONSORT2010 Statement updated guidelines for reporting parallel group randomized trials.^14^

### 2.2 Inclusion and exclusion criteria

For this study, we included all participants with a completed questionnaire 3 years after the first CS. Participants underwent a first CS as part of the 2Close study. Patients with gynecological symptoms before the CS or known causes of menstrual disorders, spotting, or cervical pathology before the CS were excluded. Also, patients with major previous surgery of the myometrium or uterine cavity (such as laparoscopic or laparotomic fibroid resection or a septum resection) were excluded. For all inclusion and exclusion criteria, see the published protocol of Stegwee et al.^12^ Pregnant and breastfeeding patients or those using hormonal contraception at 3 years follow‐up were excluded from the current analyses, based on the criteria required for the diagnosis of CSDi.^7^

### 2.3 Data collection

All participants were invited for a TVUS as part of the 2Close study 3 months after their CS. During this TVUS, a structural assessment of the uterus, uterine scar, and niche was performed by an independent ultrasonographer using the standardized criteria earlier described by Jordans et al.^8^ A uterine niche is defined as an indentation at the site of the CS scar with a depth of at least 2 mm in the sagittal plane. In the sagittal plane, length, depth, residual myometrium, and adjacent myometrial thickness were measured. In the transverse plane, the width of the niche was evaluated. Gel infusion sonohysterography (GIS) or saline infusion sonohysterography (SIS) was recommended if there was no fluid present in the niche or uterine cavity. If there was already fluid or the niche was easily recognized, it was not needed to do this. If a uterine niche was not directly visible on ultrasound, a gel GIS was performed to analyze the uterine scar.

Three years after their CS, the participants received a digital questionnaire to discuss menstruation characteristics, dysmenorrhea (Numeric Rating Scale), Quality of Life (using Short‐Form‐36^15^), and the use of medical and/or surgical therapy because of gynecological symptoms. This questionnaire discussed all symptoms, except odor associated with abnormal blood loss. All symptoms were self‐reported; no additional information from healthcare providers was acquired.

### 2.4 Outcomes

Our primary outcome was to determine the prevalence of patients who met the criteria for CSDi 3 years after their CS. The diagnosis of CSDi was based on the presence of one primary symptom or at least two secondary symptoms, all in the presence of a sonographic finding of a niche (i.e., an indentation at the site of the CS scar with a depth of at least 2 mm).^7^, ^8^

Secondary outcomes were the prevalence of primary and secondary symptoms in patients with a uterine niche. Additionally, we reported on the prevalence of CSDi in those who had undergone a second CS during the follow‐up period.

The primary and secondary symptoms of CSDi are defined in Table 1.

**TABLE 1:** Definition of primary and secondary symptoms of cesarean scar disorder.

Symptoms	Definition
*Primary symptoms of CSDi*	
Postmenstrual spotting	At least 2 days of postmenstrual spotting after the menstruation ended
Pain during uterine bleeding	Menstruation with an NRS score of 5 or higher and the pain is worse since the CS
Technical issues with catheter insertion during embryo transfer	Problems with the catheter during ART due to the CS scar
Secondary unexplained infertility combined with intrauterine fluid	Failure to achieve a pregnancy after 12 months or more of regular unprotected sexual intercourse and the presence of intrauterine fluid during TVUS
*Secondary symptoms of CSDi*	
De novo dyspareunia	Pain during sexual intercourse that is new since the CS
Abnormal vaginal discharge	Vaginal fluid loss other than normal vaginal discharge
Chronic pelvic pain	Lower abdominal pain besides during menstruation for more than 3 months
Avoiding sexual intercourse	Avoiding sexual intercourse due to spotting, uterine pain, or blood loss
Secondary unexplained infertility	Failure to achieve a pregnancy after 12 months or more of regular unprotected sexual intercourse or need for ART to become pregnant while the first pregnancy was after natural conception
Secondary unexplained infertility despite ART	Failure to achieve pregnancy despite ART
Negative self‐image	Having a negative feeling about your self‐image (≤3 on a scale 1–6)
Discomfort during participation in leisure activities	Experiencing difficulty doing normal leisure activities due to spotting, uterine pain, or blood loss

Abbreviations: ART, assisted reproductive technology; CS, cesarean section; CSDi, cesarean scar disorder; NRS, numeric rating scale; TVUS, transvaginal ultrasound.

### 2.5 Statistical analyses

Demographic data were presented as n (%) for categorical variables and mean (SD) or median (interquartile range, IQR) for continuous variables. Statistical significance was defined as a *p* < 0.05. Independent *t*‐test was used to calculate differences in baseline characteristics. Data were analyzed in SPSS version 28.

The prevalence of CSDi in our study population was calculated using the number of unique individuals who met the criteria for the disorder compared to the total group of patients who met the inclusion criteria.

## 3 RESULTS

**FIGURE 1:**
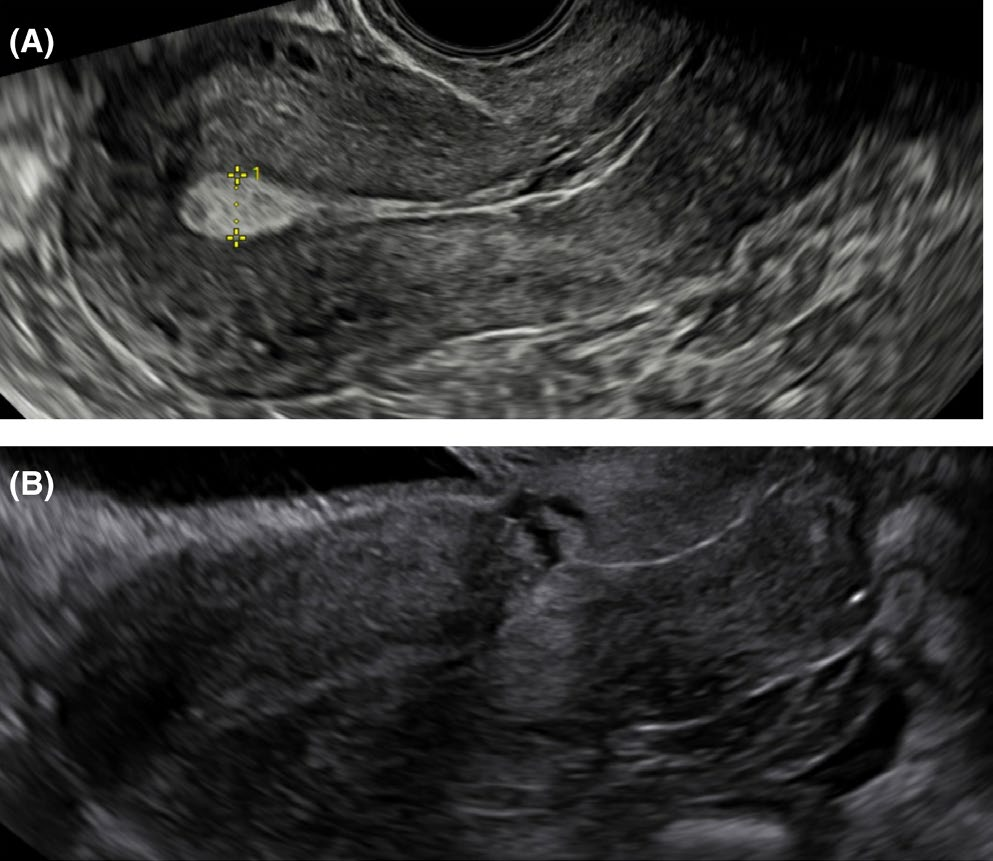
(A) An uterus 3 months post section without a uterine niche. (B) An uterus 3 months post section with a uterine niche.

The original 2Close study included 2292 participants. Three months after their CS, 71.2% of the participants had a uterine niche visible on ultrasound^5^ (Figure 1).

**FIGURE 2:**
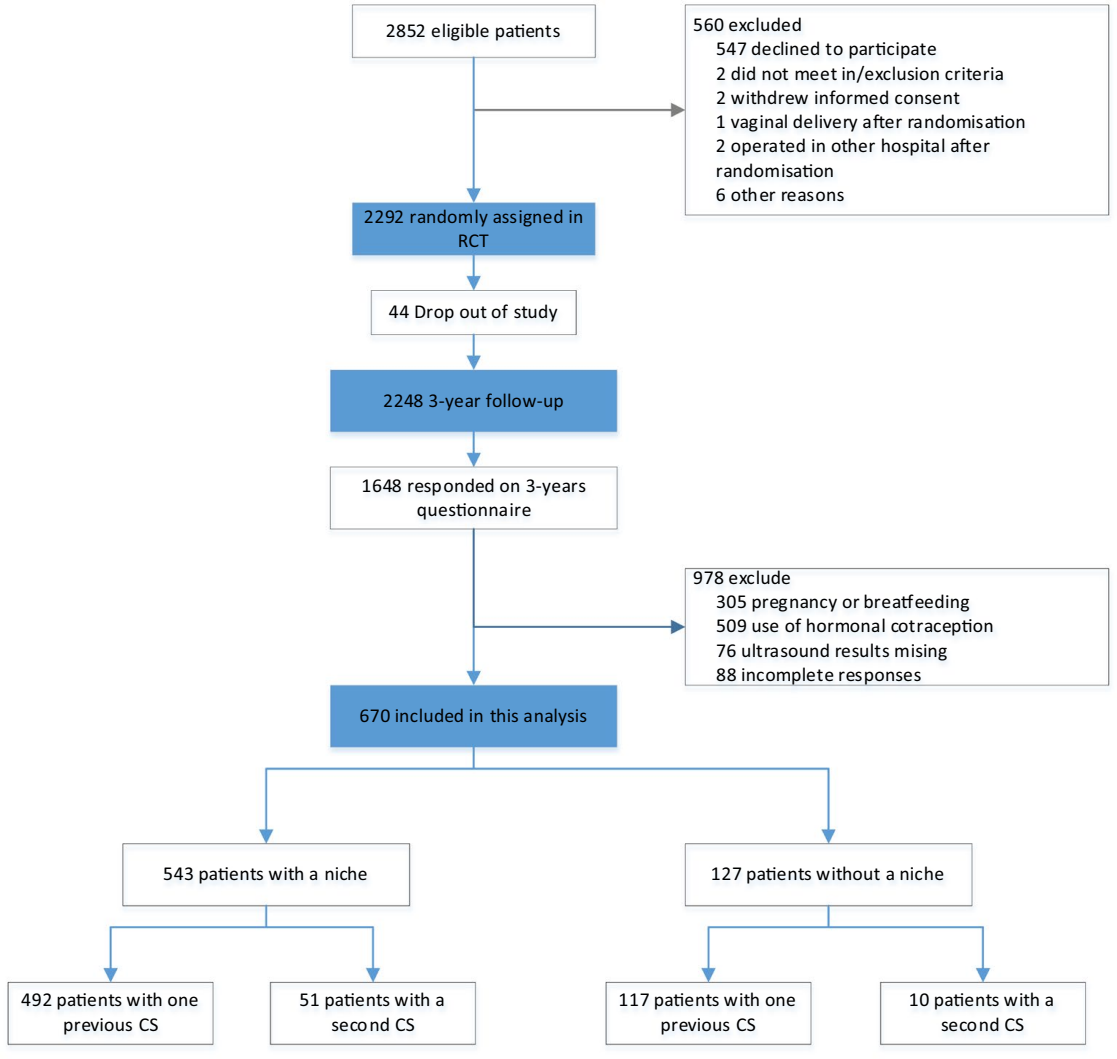
Flowchart summarizing selection of included population.

A total of 1648 patients responded to the 3‐year questionnaire (response rate 73.3%). We applied the following exclusions: pregnancy or breastfeeding (*n* = 305), use of hormonal contraception (*n* = 509), missing ultrasound (*n* = 76), and incomplete responses (*n* = 88). This left 670 patients who were included in the current analysis; 543 (81.0%) had an ultrasound finding of a uterine niche and 127 (19.0%) did not have a uterine niche (Figure 2). During the follow‐up, 609 (90.9%) patients had one CS and 61 (9.1%) patients had undergone a second CS. The baseline characteristics of the patients included are presented in Table 2.

**TABLE 2:** Baseline characteristics.

	Patients with a niche (*n* = 543)	Patients without a niche (*n* = 127)
Age, mean (SD), years	36.0 (4.6)	36.3 (4.5)
BMI, mean (SD)	26.8 (4.9)	26.3 (4.4)
Number of pregnancies achieved since CS, no. (%)	1.2 (0.4)	1.1 (0.3)
Undergone a second CS during follow‐up, no. (%)	51 (9.4)	10 (7.8)
Niche volume, median (IQR), mm^3^	*n* = 542 20.9 (8.9–51.4)	NA
RMT, mean (SD), mm	6.2 (3.0)	NA
Large niche prevalence (RMT ≤3 mm), No. (%)	77 (14.2)	NA

Abbreviations: 95% CI, 95% confidence interval; BMI, body mass index; CS, cesarean section; *N* represents the number of patients with data available; NA, not applicable; RMT, residual myometrium thickness.

The prevalence of CSDi was 42.5% (285/670); 202 patients (30.1%) met the criteria for at least one primary symptom, and 155 patients (23.1%) met the criteria for two or more secondary symptoms. In total, 285 unique individuals (42.5%) were diagnosed with CSDi. The prevalence of the symptoms in patients with a uterine niche (*n* = 543) is shown in Table 3.

**TABLE 3:** Prevalence of primary and secondary symptoms in patients with a niche, *n* (%).

Primary symptoms	Prevalence, no. (%)	Secondary symptoms	Prevalence, no. (%)
Postmenstrual spotting	178 (32.8)	De novo dyspareunia	89 (16.4)
Pain during uterine bleeding	50 (9.5)	Abnormal vaginal discharge	126 (23.2)
Technical issues with catheter insertion during embryo transfer	4 (0.7)	Chronic pelvic pain	190 (35.0)
Secondary unexplained infertility combined with intrauterine fluid	1 (0.2)	Avoiding sexual intercourse	25 (4.6)
NA	NA	Secondary unexplained infertility	78 (14.4)
NA	NA	Secondary infertility despite ART	1 (0.2)
NA	NA	Negative self‐image	27 (5.0)
NA	NA	Discomfort during participation in leisure activities	33 (6.1)

Abbreviations: ART, assisted reproductive technology; NA, not applicable.

In the follow‐up period, 61 patients in our population underwent a second CS and 24 patients (39.3%) met the criteria for CSDi. Seventeen (27.9%) had a primary symptom and seven (11.5%) had two or more secondary symptoms.

## 4 DISCUSSION

In this secondary analysis of a multicenter RCT, we found a high prevalence of CSDi 3 years after the first CS. The most frequently reported symptoms following CS were chronic pelvic pain (35.0%), postmenstrual spotting (32.8%), and abnormal vaginal discharge (23.2%). These findings provide valuable insights into the potential consequences of CS and warrant further attention in clinical practice and research. Additionally, it will help with counseling for patients scheduled for a CS.

This is the first study focusing on the prevalence of CSDi, with a structured based list of symptoms. While various systematic reviews have been published in the literature on symptoms related to the presence of a niche, a wide array of definitions exists for both the uterine niche itself as well as the symptoms associated with it.^9^, ^16^, ^17^, ^18^, ^19^, ^20^

This study is a secondary analysis of the long‐term results of the 2Close study. The primary goal of the 2Close study was to evaluate single‐ versus double‐layer uterine closure at CS in relation to niche development and postmenstrual spotting. At 9 months and 3 years follow‐up, there was no difference between these closure techniques for either of these outcomes.^5^, ^13^ The large niche prevalence at 3 months postoperative was 35.6% in the single‐layer closure group compared to 35.1% in the double‐layer closure group, with a risk difference of 0.98 (95% CI: 0.87–1.11). Additionally, none of the examined menstrual outcomes was significantly different between the closure techniques at 9 months and 3 years follow‐up. Based on these results, we decided it was justified to merge the data from the two groups with different suture techniques for the current analysis.

In the population included in this study, we have a relatively high prevalence of a niche (81.0%), compared to the absence of a niche at 3 months follow‐up. Other studies report a niche prevalence of 42%–84% in a random population after CS using GIS or TVUS 6–12 months after CS.^9^, ^10^, ^11^, ^21^, ^22^, ^23^ The prevalence differed based on niche definition and the imaging technique used. An explanation for the observed difference in prevalence seen in our study could be attributed to patient selection. In our analysis, we excluded patients who were pregnant or breastfeeding. It is known that a niche could influence fertility and that patients without a niche tend to have better chances of achieving a pregnancy.^24^, ^25^ In addition, patients with a niche may be more willing to respond to the questionnaire at a 3‐year follow‐up. Therefore, it is likely that we inadvertently excluded patients without a niche from our analysis, which could have led to an overrepresentation of patients with a niche and consequently an increased apparent prevalence of CSDi. On the other hand, we also excluded patients who were using hormonal contraception. We are aware that patients with a symptomatic niche are more likely to use hormonal contraception as therapy for niche related symptoms. The exclusion of both groups may have introduced selection bias that needs to be considered when interpreting the results of our study.

While determining the prevalence of CSDi is relevant, it is important to approach the results of this study with caution. We acknowledge that there may be a potential underestimation of fertility‐related symptoms due to the 3‐year interval following the participants' CS. It is possible that after additional years, more patients may have tried to conceive without success. Additionally, we did not evaluate for the symptom of “*odor associated with abnormal blood loss*,” which was a secondary symptom of CSDi. In the literature, only Szafarowska et al. reported the prevalence of this symptom in patients with a niche.^26^ They reported that eight out of 85 patients (9.4%) were suffering from odor associated with abnormal blood loss and underwent a diagnostic or operative hysteroscopy for this complaint. Adding this symptom to the questionnaire would likely have increased the prevalence of CSDi in our population, further confirming the importance of this condition. Our study is a starting point with the available evidence at this moment and future studies should be developed to gain more information about the prevalence of CSDi.

In our study, the majority of the population had undergone one prior CS, while only 9.1% had a second CS. Among this group, the prevalence of CSDi was similar to that of the group with only one prior CS. However, it is important to note that we did not perform a new TVUS examination after the second CS. Therefore, it is probable that more patients developed a uterine niche after their second CS, as studies have shown that the likelihood and volume of a niche will increase with the number of CSs.^9^, ^27^ It is worth mentioning that larger niches with a higher volume are associated with an increased risk of postmenstrual spotting.^16^ Furthermore, the interval between the second CS and administration of the questionnaire was relatively short, which may have limited the reporting of some symptoms such as infertility or difficulties in ART treatment. Consequently, in populations comprising patients with multiple CSs, we would expect to find a higher prevalence of CSDi; however, perhaps a longer follow‐up duration is needed.

CSDi is an iatrogenic disorder as a consequence of CS. Currently, it is not addressed in the counseling guidelines regarding CS.^28^, ^29^ However, with the emergence of the findings in this study, it is advisable to incorporate this information into the counseling process for patients who are scheduled for a CS or to discuss it with them afterward. Many patients will unknowingly suffer with a symptomatic cesarean scar niche which is known to have a negative impact on quality of life.^30^ Recognition of the high prevalence of CSDi can help diagnose the condition and help patients obtain appropriate treatments.^31^

In the future, prospective cohort studies should be conducted to examine the long‐term consequences and prevalence of CSDi following a CS in comparison with these symptoms after a vaginal delivery or in patients without a uterine niche. This information would also be valuable for validation of the symptoms included in the definition of CSDi. It would be interesting to analyze the difference in prevalence of these symptoms for women with and without a uterine niche. For these studies, we recommend evaluating the uterus 6 months post‐surgery for niche presence using a proper GIS, following the protocol described by Jordans et al.^8^ These studies should also prioritize investigating the influence of each individual symptom on the quality of life for patients with and without CSDi. These valuable data will aid in validating the symptoms associated with CSDi and may even lead to adjustments in diagnostic criteria. Our advice will be to conduct a minimal follow‐up of 5 years for reproductive symptoms and to include patients with one or more CSs.

The major strengths of our study are the large database of patients with one previous CS, the performance of a TVUS 3 months after the CS, and the high response rate (73.3%) to the questionnaire administered 3 years after surgery. However, the optimal timing for evaluating the uterine scar following surgery is debatable. A study by van der Voet et al. showed a decrease in the RMT measurement in the first year after a CS compared to RMT measured 12 weeks after CS.^32^ However, the prevalence of a niche and niche depth remained the same over time. There is no universal guideline regarding the timing of uterine scar assessment after a CS. Unfortunately, performing another ultrasound examination on all patients was not logistically feasible at the 3 years follow‐up.

The most important limitation of our study is the selection of the participants included in this study. Our analysis included only 29% of the 2248 patients who were included in the 3‐year follow‐up. Our population has an almost 10% higher percentage of patients with a uterine niche compared to the original 2Close study group (81.0% vs. 71.2%). Based on our analysis, the actual incidence of CSDi likely falls within a range of 12.7% (285 out of the total included 2248 patients in the 2Close study) to 42.5% (285 out of 670 included patients in this analysis), with the most probable estimate closer to the higher end of this range. Another limitation is that this study only focused on women with a uterine niche, however interesting would be as well to compare the prevalence of symptoms with women without a uterine niche or a vaginal delivery. Future studies, should include these groups as well.

Our results should be interpreted in the context of the study design. Most notably, all symptoms were self‐reported by participants. This introduces the possibility of recall bias, particularly regarding symptoms related to issues during assisted reproductive technology (ART), where those with infertility would be more likely to report symptoms.

## 5 CONCLUSION

This study found a high prevalence of CSDi in patients 3 years after their first CS. This prevalence should be included in the counseling of patients undergoing a CS and could be helpful to reduce the number of unneeded CSs. Future studies should be developed to further investigate the prevalence of CSDi in a broader population and to identify possible risk factors for developing this disorder.

## AUTHOR CONTRIBUTIONS

Saskia J. M. Klein Meuleman: Conceptualization, data curation, formal analysis, methodology, software, resources, and writing—original draft. Sanne I. Stegwee: Conceptualization, data curation, formal analysis, funding acquisition, investigation, methodology, project administration, software, resources, validation, and writing—review and editing. Robert A. de Leeuw: Conceptualization, funding acquisition, methodology, supervision, and writing—review and editing. Carry Verberkt: Data curation, investigation, project administration, validation, and writing—review and editing. Judith A. F. Huirne: Funding acquisition, methodology, supervision, and writing—review and editing. Thierry van den Bosch, Shunichiro Tsuji, Michal Pomorski, Jian Zhang, Tom Bourne, Ertan Saridogan, Grigoris Grimbizis, Oliver Donnez, Ally Murji, and Pere N. Barri: Supervision.

## FUNDING INFORMATION

The initial 2Close study was performed with funding from ZonMw: The Netherlands Organisation for Health Research and Development (project no. 843002605).

## CONFLICT OF INTEREST STATEMENT

Judith A. F. Huirne received grants from ZonMw for the conduct of the study, and additionally received grants from Samsung, NWO‐TTW, and PlantTec Medical GmbH, and a fee from Olympus, all outside the submitted work. An institutional grant was received from Olympus, Hologic, Benetec, and Medical Dynamics, outside the submitted work. All other authors declare no competing interests.

## ETHICS STATEMENT

The initial 2Close study was approved by the Institutional Review Board of Amsterdam UMC, location VU University Medical Centre, in December 2015 (registration no. 2015.462), and by the boards of all participating hospitals before the start of inclusion. All participants provided written informed consent before taking part in the study.
